# Measurement invariance of the depressive symptoms scale during adolescence

**DOI:** 10.1186/1471-244X-14-95

**Published:** 2014-03-31

**Authors:** Jennifer Brunet, Catherine M Sabiston, Michael Chaiton, Nancy CP Low, Gisèle Contreras, Tracie A Barnett, Jennifer L O’Loughlin

**Affiliations:** 1 School of Human Kinetics, University of Ottawa, 125 University Pr., Montpetit Hall, Room 339, Ottawa, Ontario K1N 6N5, Canada; 2 Faculty of Kinesiology & Physical Education, University of Toronto, 55 Harbord Street, Toronto, Ontario M5S 2W6, Canada; 3 Dalla Lana School of Public Health, University of Toronto, Ontario Tobacco Research Unit, T523, 33 Russell St, Toronto, Ontario M5S 2S1, Canada; 4 Department of Psychiatry, McGill University, 1033 Pine Ave West, Montreal, Quebec H3A 1A1, Canada; 5 Research Hospital Center of the University of Montreal, Department of Social and Preventive Medicine, University of Montreal, 850 Saint-Denis, S02-370, Montreal, Quebec H2X 0A9, Canada; 6 Sainte-Justine Hospital Research Center, Department of Exercise Science, Concordia University, 5757 Decelles avenue, Montreal, Quebec H3S 2C3, Canada

**Keywords:** Factorial validity, Depression, Anxiety, Sex, Longitudinal, Youth

## Abstract

**Background:**

This study examined (1) the factor structure of a depressive symptoms scale (DSS), (2) the sex and longitudinal invariance of the DSS, and (3) the predictive validity of the DSS scale during adolescence in terms of predicting depression and anxiety symptoms in early adulthood.

**Methods:**

Data were drawn from the Nicotine Dependence in Teens (NDIT) study, an ongoing prospective cohort study of 1,293 adolescents.

**Results:**

The analytical sample included 527 participants who provided complete data or had minimal missing data over follow-up. Confirmatory factor analysis revealed that an intercorrelated three-factor model with somatic, depressive, and anxiety factors provided the best fit. Further, this model was invariant across sex and time. Finally, DSS scores at Time 3 correlated significantly with depressive and anxiety symptoms measured at Time 4.

**Conclusions:**

Results suggest that the DSS is multidimensional and that it is a suitable instrument to examine sex differences in somatic, depressive, and anxiety symptoms, as well as changes in these symptoms over time in adolescents. In addition, it could be used to identify individuals at-risk of psychopathology during early adulthood.

## Background

The prevalence of major depression in North American adolescents 12 to 18 years of age ranges from 6% to 9% [1]. An even higher proportion experience depressive symptoms without meeting formal criteria for clinical diagnosis [2]. Sub-threshold depression during adolescence has been linked to major depression disorder (MDD) in adulthood [3,4]. In addition, adolescents who report higher levels of depressive symptoms report lower levels of well-being, self-esteem, physical health, and educational performance [3,5].

To facilitate investigation of the causes and consequences of sub-threshold depression, measures of depressive symptoms have been developed. Kandel and Davies [6] developed a 6-item depressive symptoms scale (DSS) to rapidly assess depressive symptoms, which has been used in a number of studies with adolescents e.g., [7-9]. It is based on the depressive mood subscale of the Hopkins Symptom Checklist SCL-90 [10], and the reliability and validity of the scores have been supported in adolescent populations [6,7,11,12]. However, only two studies to date have reported on its factor structure. Kandel and Davies [6] first tested the factor structure in a small clinical sample (*n* = 29) of adolescents and concluded that the scale was unidimensional. This finding was supported in a larger sample of secondary school students [13], but the results of the factor analysis were not reported and the authors did not test alternative models in this study. Although these two studies suggest that the DSS is unidimensional, Costello and Angold [14] suggested that the items that comprise the scale assess anxiety and somatic symptoms in addition to depressive symptoms. Specifically, the items assessing feelings of nervousness and worry are characteristic of anxiety, the items assessing feelings of fatigue and insomnia are characteristic of somatic symptoms of both depression and anxiety, and the items assessing feelings of sadness and hopelessness are characteristics of depression [15]. Therefore, alternative factor structures of the DSS should be tested.

Conceptually, there is disagreement regarding whether depression and anxiety are distinct constructs [16,17], or whether they are two facets of a single syndrome since they often co-occur and there is overlap in their symptoms [18]. Clark and Watson [19] developed the tripartite model which has been supported in youth populations [17], which posits that the affective dimensions of distress can be conceptualized as either general affective distress or as two specific factors (i.e., depression and anxiety). In addition to conceptual uncertainty, factor-analytic studies of other measures that claim to assess depressive symptoms raise questions about the dimensionality of the DSS. In particular, varying factor structures have been reported for other self-report measures including the Hospital Anxiety and Depression Rating Scale (HADS) [20], the Beck Depression Inventory – Second Edition (BDI-II) [21], the Center for Epidemiological Studies Depression scale (CES-D) [22], and the Reynolds Adolescent Depression Scale (RADS) [23]. For example, researchers have found support for both a two-factor model and a three-factor model for the HADS [24,25] and the BDI-II [26,27]. Others report evidence for a four-factor model for the CES-D [28,29]. Based on these conceptual and methodological perspectives, it is possible that the DSS may be best conceptualized as multidimensional, with three related factors (i.e., somatic, depressive, and anxiety symptoms). As well, considering that it can be difficult to determine if somatic complaints are expressions of psychological distress as they may be attributed to biological and hormonal changes associated with puberty (e.g., hormonal fluctuations during menstrual cycles can cause fatigue, growth spurts can bring changes in appetite), and that somatic symptoms during adolescence have been shown to predict depression and other mental disorders during adulthood [30], it may be appropriate to distinguish between somatic and affective symptoms in a two-factor model.

In addition, although other measures of depression have been shown to possess evidence of sex and longitudinal invariance e.g., [28,29], evidence for the measurement invariance for the DSS are lacking since such tests have yet to be conducted. Invariance refers to the extent to which scores on a measure hold equivalent meaning across groups or conditions [31]. Given that other self-report measures of depression have been found to be non-invariant across sex [32,33] and that differences in depressive symptoms by sex and over time are of interest to researchers e.g., [11,34], conducting invariance testing with the DSS is critical. If shown to be invariant, reported differences in levels of depressive symptoms across these characteristics can be interpreted as true differences rather than measurement artifact [35].

Finally, demonstrating that the DSS predicts future psychopathology has important practical implications because depressive symptoms during adolescence have been linked to MDD and anxiety in adulthood [3,4]. Specifically, the scale could be used to identify which individuals should be targeted for intervention during adolescence to prevent future MDD. Kandel and Davies [13] showed that levels of depressive symptoms assessed during adolescence were significantly associated with depressive symptoms in young adulthood. However, evidence regarding the predictive validity of the DSS is limited to this study and only using a global DSS score. If the factor structure of this measure is multidimensional, it will be important to determine whether the different subscales allow researchers to predict future mental health. Adolescents in community samples who are diagnosed with an anxiety disorder can develop depression, while a smaller proportion of those diagnosed with depression later develop anxiety [36,37]. Therefore, the specific subscales of the DSS may be more accurate (i.e., valid and reliable) predictors of future specific psychopathological symptoms than a global symptoms scores, and thus the probability of accurately identifying adolescents at risk of developing specific psychopathological symptoms may be increased. This highlights the need to examine the predictive validity of the multidimensional factor structure if this conceptualization holds and is shown to be superior to a unidimensional factor structure.

The current investigation had three objectives. The first objective was to test the factor structure of the DSS in a large, non-clinical sample of adolescents by comparing a single-factor, an intercorrelated two-factor, and an intercorrelated three-factor model. Based on a content review of the scale, reports that the scale may be multidimensional [14], and theoretical postulations about how anxiety and depression may interrelate [19], it was hypothesized that an intercorrelated three-factor model would demonstrate the best fit. The second objective was to examine sex and longitudinal invariance of the measurement parameters for the best fitting model. It was hypothesized that the measure would demonstrate sex and longitudinal invariance. The third objective was to test the validity of the DSS (or DSS subscales) in predicting future depression and anxiety. It was hypothesized that the scale (or subscales) assessed during adolescence would show significant associations of moderate magnitude with depression and anxiety disorders assessed during young adulthood.

## Methods

### Procedures and participants

Data were drawn from the Natural History of Nicotine Dependence in Teens (NDIT) study, a longitudinal cohort investigation of 1,293 adolescents who were 12-13 years at baseline [38]. The NDIT study was designed to investigate the natural course of nicotine dependence, as well as identify genetic, sociodemographic, psychosocial, and environmental risk factors for the onset of smoking and nicotine dependence. Data were also collected on health behaviors at each survey cycle, and data on anthropometrics and blood pressure were collected at baseline and biannually thereafter to allow for the investigation of other risk factors for cancer and cardiovascular disease. Participants were recruited from all grade 7 classes in a convenience sample of 10 secondary schools in Montreal, Quebec, selected to represent a range of socioeconomic status (low, medium, high), geographic locations (urban, suburban, rural), and languages [French (n = 3), English (n = 7)]. French or English questionnaires were administered according to the language used in each study school. If questionnaire items were already available in French, these items were used. If the items were not available in French, they were translated by two Francophone physicians, back-translated to test the accuracy of the translation, and then pilot tested in the target group for readability and ease of comprehension. The study received approval from the McGill University Institutional Review Board and the Centre de Recherche du Centre Hospitalier de l’Université de Montréal. Participants provided assent and a parent/guardian provided written informed consent.

The baseline questionnaire was administered in the fall of 1999. The interval between each subsequent survey cycle was 3-4 months, resulting in 20 survey cycles over the five years during secondary school. At each of these 20 survey cycles, participants completed a self-administered questionnaire during regular class time. An additional data collection took place in 2007-2008 (survey cycle 21), when participants were aged 18-24 years, using mailed self-report questionnaires. Data from survey cycles 1 (Time 1), 10 (Time 2), 19 (Time 3), and 21 (Time 4) were used in the current analyses when the mean (*SD*) ages of participants were 12.6 (0.4), 14.9 (0.4), 17.1 (0.4), and 20.2 (0.6) years, respectively.

The sample for the analyses involving Time 1, 2, and 3 (used to address objectives 1 and 2) comprised 527 participants (46.3% boys) who provided complete data or had less than 20% of their data missing by design or at random at each of Time 1, 2, and 3. This sample was reduced to 426 participants (44.9% boys) for the analyses involving Time 4 (used to address objective 3) due to attrition. No statistically significant differences were observed in baseline depressive symptoms scores (*F* = 2.24, *df* = 1, *p* > 0.05) or sex (χ^2^ = 1.38, *df* = 1, *p* > 0.05) between adolescents included and not included in the analyses. Also, the percentage of mothers (48.3% vs. 41.9%, χ^2^ = 3.77, *df* = 1, p > 0.05) and fathers (48.5% vs. 44.0%, χ^2^ = 1.75, *df* = 1, p > 0.05) who attended university did not differ significantly between the retained and not-retained groups. However, participants included in the analyses were younger (mean = 12.1, *SD* = 0.7 vs. mean = 12.4, *SD* = 0.4; *F* = 70.54, *df* = 1, *p* < 0.001), and more likely to have completed the questionnaire in English (75.1% vs. 57.1%, χ^2^ = 42.40, *df* = 1, *p* < 0.05), than participants excluded from the analyses. Data on ethnicity collected at Time 4 indicated that 77.5% of participants self-identified as White.

### Measures

Participants completed the 6-item DSS [6] at Time 1, 2, and 3. Participants were asked to indicate the degree to which they (1) “felt too tired to do things”, (2) “had trouble going to sleep or staying asleep”, (3) “felt unhappy, sad, or depressed”, (4) “felt hopeless about the future”, (5) “felt nervous or tense”, and (6) “worried too much about things.” Participants responded to each item using a 4-point Likert scale (modified from the original 3-point scale) ranging from 1 (“*never*”) to 4 (“*often*”). Because of the NDIT study design, the recall period was the past 3 months. Researchers have reported test–retest reliability (intraclass correlation coefficients; ICC) > 0.76, internal consistency coefficients (Cronbach’s α) > 0.72, and face validity for the DSS in previous studies with adolescent populations [6,7]. Also, the correlation coefficient (Pearson *r* = 0.72) previously reported between DSS scores and scores on the depression subscale of the SCL-90 in a clinical sample of youth 14-16 years of age provides support for the concurrent validity of this scale [6].

The Major Depression Inventory (MDI) [39] was used in survey cycle 21 (Time 4) to assess depressive symptoms. Participants were asked to self-report the frequency with which they experienced 12 symptoms (e.g., “felt that life wasn’t worth living”, “suffered from reduced appetite”) in the past 14 days using a 6-point response scale ranging from 0 (“*at no time*”) to 6 (“*all the time*”). A total MDI score was calculated and symptom-specific subscale scores were calculated for somatic symptoms and affective/cognitive symptoms, with higher scores indicating more frequent depressive symptoms. Scores on the MDI have been shown to be valid and reliable in adults [40,41].

Lifetime presence of symptoms of panic disorder, social phobia, agoraphobia, and generalized anxiety disorder was assessed at Time 4. Participants indicated whether they had ever experienced each symptom by indicating 1 (“*no*”) or 2 (“*yes*”). Panic disorder symptoms were present if participants responded “yes” to experiencing “an attack of fear or panic when all of a sudden you felt very frightened, anxious or uneasy” and/or “an attack when all of a sudden, you became dizzy, very uncomfortable, short of breath, dizzy, nauseous, your heart pounded, or you thought that you might lose control, die or go crazy.” Social phobia disorder symptoms were present if participants responded “yes” to experiencing “a time when you felt very afraid or really shy meeting new people, going to parties, going on a date” and/or “a time when you felt very afraid or uncomfortable when you had to do something in front of a group of people (giving a speech, speaking in class).” Agoraphobia disorder symptoms were present if participants responded “yes” to experiencing “a time in your life when you felt afraid of being in crowd, going to public places, traveling alone” and/or “a time in your life when you became very upset or nervous in crowds, public places, or traveling.” Generalized anxiety disorder symptoms were present if participants responded “yes” to experiencing “a time when you were a “worrier” (when you worried a lot more about things than other people with the same problems)” and/or “a time when you were much more nervous or anxious than most other people with the same problems.” An additional anxiety variable was created whereby participants who reported at least one of the above anxiety disorder symptoms were coded as yes for ‘presence of anxiety’.

Questions on anxiety screening in survey cycle 21 (Time 4) were based on the Canadian Community Health Survey (CCHS; Cycle 1.2) [42]. The CCHS interview is based on the World Mental Health Survey Initiative Version of the World Health Organization (WHO) Composite International Diagnostic Interview (CIDI) [43]. While the CCHS and the CIDI are interviewer-administered, the NDIT questionnaire was self-administered since the interviewer-administered format was neither feasible nor desirable since social desirability bias is lower with the self-administered than interviewer-administered version [44]. Further, there is acceptable agreement between the self- and interviewer-administered versions of the CIDI [45,46]. Of note, the question wording and instructions, response categories, formatting, question order, and response categories of the interviewer-administered CCHS were preserved.

### Data analysis

#### Factor structure

Multiple imputation expectation-maximization algorithm [47] was used to estimate and replace missing observations when data loss on the DSS was minimal (i.e., 0.2%-2.3%). The factor structure of the DSS was tested at each time point using confirmatory factor analysis (CFA) with robust maximum likelihood estimation in LISREL (version 8.80) following recommended procedures [35]. Owing to the ordered-categorical nature of the data, the Satorra-Bentler scale chi-square statistic and robust standard errors were used since they yield unbiased goodness-of-fit indices when dealing with non-normal data. Three models were tested: (i) a single-factor structure where all items on the DSS were specified to represent one latent variable termed generalized distress, (ii) a two-factor intercorrelated structure where the items represent two latent variable: somatic symptoms and generalized affective distress, and (iii) a three-factor intercorrelated structure where the items represent three latent variables: somatic symptoms, depressive symptoms, and anxiety symptoms. Table 1 presents the items corresponding to the latent variables in each model. The factor loading for the first item of each latent variable was set to 1.0 to establish the metric of the latent variable.

**Table 1:** Standardized factor loadings of the 6-items of the depressive symptoms scale

	**Single-factor model**	**Two-factor model**		**Three-factor model**		
**Items**	**I**	**I**	**II**	**I**	**II**	**III**
1. Felt too tired to do things	.37/.63/.66	.54/.74/.81		.54/.74/.82		
2. Had trouble going to sleep or staying asleep	.35/.60/.57	.50/.70/.69		.51/.71/.70		
3. Felt unhappy, sad, or depressed	.62/.81/81		.62/.81/.81		.68/.87/.87	
4. Felt hopeless about the future	.60/.76/.71		.60/.77/.72		.66/.81/.75	
5. Felt nervous or tense	.58/.73/.82		.59/.73/.82			.70/.78/.84
6. Worried too much about things	.74/.81/.86		.75/.81/.87			.81/.88/.90

Note. First, second, and third factor loadings correspond to Time 1, 2, and 3, respectively.

Based on recommendations by Chen [48] for comparing nested models, the single-factor, intercorrelated two-factor, and intercorrelated three-factor models were compared at each time point by examining change in Comparative Fit Index (CFI) and Root Mean Square Error of Approximation (RMSEA) values (∆CFI ≤ 0.010 combined with ∆RMSEA ≤ 0.015 indicates no significant differences between models), as well as observation of multiple robust indices [49], namely the CFI, RMSEA, and Standardized Root Mean Square of the Residuals (SRMR) values. Values of 0.90 and higher for the CFI indicate acceptable fit of the model, and values lower than 0.080 for the RMSEA and SRMR indicate acceptable model fit [49]. The strength of the standardized factor loadings between each indicator and its corresponding latent variable were also examined.

#### Invariance

The sex and longitudinal invariance of the best fitting DSS model was tested in a multi-group CFA framework [35], which consisted of testing different levels of invariance by comparing nested models [31]. The first step involved testing a baseline model (Model 1) with no constraints that was estimated simultaneously across groups (i.e., across sex; across time whereby all three measurements were entered into one model). This step serves to test the weakest level of measurement invariance and represents “configural invariance.” Invariance at this step would suggest that the pattern of fixed and free factor loadings is invariant across groups. The second step involved adding constraints on the factor loadings to be equal across groups (Model 2). This is a stronger test of factorial invariance and serves to test for “metric invariance” to ensure that the expected change in the observed score on the DSS per unit change on the latent variable is equal across groups. The third step involved adding equality constraints on the item intercepts across groups and represents “scalar invariance” (Model 3). The fourth step involved adding equality constraints on the uniquenesses (i.e., disturbances) associated with the items across groups (Model 4). This is a “strict” test of invariance and is akin to testing invariance of the reliability associated with the items if the latent factor variances are equal. Additional tests of invariance were conducted by imposing equality constraints across groups on factor variances (Model 5), factor covariances (Model 6; only if the best fitting model was the two- or three-factor model), and latent factor means (Model 7) [31]. Invariance at each step was established if a ∆CFI ≤ 0.010 was supplemented by a ∆RMSEA ≤ 0.015 [48]^a^.

#### Predictive validity

To assess the final objective of this study (i.e., the predictive validity of the best fitting model), correlations (Pearson and Point-Biserial) were computed between the DSS scores obtained at Time 3 and depression and anxiety disorders as assessed with the MDI and CIDI at Time 4.

## Results

### Factor structure

The fit indices for all three CFA models are presented in Table 2. The intercorrelated three-factor model provided a better fit to the data at Time 1, 2, and 3 based on the standardized factor loadings (Table 1), and fit indices (Tables 1 and 2). The Cronbach’s alpha coefficients for this model were > 0.70 at each time point, except for the somatic and depressive symptoms factors at Time 1 (α ≥ 0.60). The scores showed moderate stability across time (ICC: Time_1-2_: = 0.54; Time_2-3_: = 0.77). Positive correlations of moderate to high magnitude were observed between the latent factors at each time point: *r*_somatic, depression_ = 0.65, 0.76, 0.77; *r*_somatic, anxiety_ = 0.59, 0.78, 0.74; *r*_depression, anxiety_ = 0.81, 0.82, 0.88 at Time 1, 2, and 3, respectively.

**Table 2:** Goodness of fit statistics for the measurement models at each time point

**Model**	**χ**^**2**^	***df***	**CFI**	**|∆CFI|**	**RMSEA**	**|∆RMSEA|**	**SRMR**
Time 1							
Single-factor	35.727	9	.965	-	.075	-	.041
Two-factors*	21.426	8	.982	.017	.057	.018	.028
Three-factors*	6.972	6	.999	.017	.018	.039	.016
Time 2							
Single-factor	119.231	9	.953	-	.153	-	.049
Two-factors*	88.627	8	.967	.014	.138	.015	.036
Three-factors*	13.522	6	.997	.030	.049	.089	.017
Time 3							
Single-factor	107.229	9	.959	-	.144	-	.049
Two-factors*	55.790	8	.981	.022	.107	.037	.027
Three-factors*	13.490	6	.997	.016	.049	.058	.015

*Note*. χ^2^ = Chi-square; *df* = degrees of freedom; RMSEA = root mean square error of approximation; CFI = confirmatory fit index; SRMR = standardized root mean squared of the residuals.

*indicates statistically significant difference between nested models based on ∆CFI > |.010| supplemented by a ∆RMSEA > |.015|.

### Sex invariance

Table 3 displays the results of the invariance testing for the intercorrelated three-factor model. It shows that the configural, metric, and scalar invariance of the model was supported, and that the factor variances and covariances were invariant across sex at each time point based on ∆CFI and ∆RMSEA values. However, the uniquenesses (i.e., disturbances) associated with each item were non-invariant at Time 3. In addition, the latent factor means were non-invariant at Time 2 and 3, indicating that there are significant differences across sexes at these time points on the DSS subscale scores, with girls reporting higher scores at both time point.

**Table 3:** Fit indices for the analyses testing sex invariance for the intercorrelated three-factor model

**Model**	**χ**^**2**^	***df***	**CFI**	**|∆CFI|**	**RMSEA**	**|∆RMSEA|**	**SRMR**
Time 1							
Configural invariance	14.287	12	.997	-	.027	-	.025
Metric invariance	16.354	15	.998	.001	.019	.005	.030
Scalar invariance	26.507	18	.989	.009	.033	.017	.030
Invariant uniquenesses	28.011	24	.995	.006	.025	.008	.035
Invariant factor variances	35.900	27	.988	.003	.035	.010	.053
Invariant factor covariances	41.375	30	.984	.004	.038	.003	.053
Invariant latent means	49.462	33	.976	.008	.044	.006	.054
Time 2							
Configural invariance	23.184	12	.995	-	.059	-	.016
Metric invariance	23.881	15	.995	0	.063	.004	.018
Scalar invariance	46.069	18	.986	.009	.077	.014	.032
Invariant uniquenesses	74.244	24	.972	.014	.089	.012	.044
Invariant factor variances	83.204	27	.970	.002	.089	0	.093
Invariant factor covariances	86.952	30	.969	.001	.085	.004	.088
Invariant latent means*	140.193	33	.940	.029	.111	.026	.096
Time 3							
Configural invariance	15.308	12	.998	-	.032	-	.023
Metric invariance	15.716	15	1.00	.002	.018	.014	.023
Scalar invariance	23.941	18	.997	.003	.036	.018	.025
Invariant uniquenesses*	68.818	24	.977	.020	.084	.048	.059
Invariant factor variances	72.597	27	.977	0	.080	.004	.063
Invariant factor covariances	90.318	30	.969	.008	.088	.008	.070
Invariant latent means*	181.294	33	.923	.046	.131	.043	.067

*Note*. χ^2^ = Chi-square; *df* = degrees of freedom; RMSEA = root mean square error of approximation; CFI = confirmatory fit index; SRMR = standardized root mean squared of the residuals.

*indicates statistically significant difference between nested models based on ∆CFI > |.010| supplemented by a ∆RMSEA > |.015|.

### Longitudinal invariance

The configural, metric, and scalar invariance of the model including measurements at all three time points was also supported, as was the invariance of the item uniquenesses, factor variances, covariances and means based on ∆CFI and ∆RMSEA values (Table 4).

**Table 4:** Fit indices for the analyses testing longitudinal invariance for the intercorrelated three-factor model

**Model**	**χ**^**2**^	***df***	**CFI**	**|∆CFI|**	**RMSEA**	**|∆RMSEA|**	**SRMR**
Configural invariance	127.124	30	.982	-	.077	-	.015
Metric invariance	135.244	33	.980	.002	.077	0	.031
Scalar invariance	191.889	42	.971	.009	.082	.005	.033
Invariant uniquenesses*	232.535	48	.978	.007	.084	.002	.042
Invariant factor variances	237.569	51	.963	.015	.083	.001	.107
Invariant factor covariances	246.727	54	.963	0	.084	.001	.121
Invariant latent means	309.438	60	.954	.009	.085	.001	.122

*Note*. χ^2^ = Chi-square; *df* = degrees of freedom; RMSEA = root mean square error of approximation; CFI = confirmatory fit index; SRMR = standardized root mean squared of the residuals.

*indicates statistically significant difference between nested models based on ∆CFI > |.010| supplemented by a ∆RMSEA > |.015|.

### Predictive validity

As reported in Table 5, Time 3 DSS subscales scores had low to moderate correlations with depressive symptoms and anxiety assessed at Time 4. Time 3 somatic symptoms had a higher correlation with Time 4 somatic symptoms than with the Time 4 affective/cognitive symptoms. In contrast, Time 3 depressive symptoms and anxiety symptoms had higher correlations with Time 4 affective/cognitive symptoms than with somatic symptoms.

**Table 5:** Correlations between Time 3 depressive symptoms scale scores and Time 4 depression and anxiety disorders scores

	**MDI**			**CIDI**				
	**Total MDI**	**Somatic**	**Affective/cognitive**	**Panic disorder**	**Generalized anxiety**	**Social phobia**	**Agoraphobia**	**Presence of anxiety**
Depressive symptoms scale								
Three-factor model								
Somatic symptoms	.43**	.43**	.37**	.28**	.22**	.16**	.11*	.21**
	(.35-.50)	(.35-.50)	(.29-.45)	(.19-.37)	(.13-.31)	(.07-.25)	(.02-.20)	(.12-.30)
Depressive symptoms	.40**	.32**	.42**	.26**	.30**	.19**	.18**	.26**
	(.32-.48)	(.23-.40)	(.34-.50)	(.17-.35)	(.21-.38)	(.10-.28)	(.09-.27)	(.17-.35)
Anxiety symptoms	.37**	.31**	.37**	.24**	.37**	.17**	.16**	.26**
	(.29-.45)	(.22-.39)	(.29-.45)	(.15-.33)	(.29-.45)	(.08-.26)	(.07-.25)	(.17-.35)

*Note.* Presence of anxiety was established if participants had at least one of the specific anxiety disorders.

*indicates significant associations at the *p* < .05 level.

**indicates significant associations at the *p* < .01 level.

## Discussion

The objectives of the current study were to test the underlying structure of the DSS in a non-clinical relatively large sample of adolescents, evaluate sex and longitudinal invariance, and examine its predictive validity. The findings support the hypothesis that the DSS can be conceptualized as a multidimensional scale, and that it meets recommended criteria to allow for accurate and meaningful comparisons across sex and time [31]. Furthermore, subscale scores on the DSS during late adolescence related to depressive symptoms and anxiety disorders in early adulthood.

Based on the current findings, the intercorrelated three-factor model fit the data best. These results are similar to previous factor-analytic studies that report multidimensional factor structures for other self-report measures including the HADS [24,25], the BDI-II [26,27], and the CES-D [28,29]. This provides support for the tripartite model [19] as well as previous research [50,51], and suggests that the DSS can be used in research as a multidimensional scale with three distinct factors related to general psychological distress including somatic, depressive, and anxiety symptoms. This conclusion differs from that of Kandel and Davies [6,13], who suggested that the DSS is unidimensional. Nonetheless, these studies differed in several important ways. First, NDIT was conducted with a large non-clinical sample of adolescents (*n* = 527), in contrast to the small clinical sample of adolescents (*n* = 29) in the earlier study [6]. Second, NDIT participants were asked to report symptoms in the last 3 months, whereas participants in Kandel and Davies’ [6,13] studies reported symptoms in the last year. Third, NDIT participants completed the scale repeatedly over time, which may have increased familiarity with the items in the scale and reduced unrelated sources of test difficulty [52]. Increasing familiarity and precision (as indicated by higher Cronbach’s coefficients over time) may have resulted in improved differentiation of the dimensions of distress in the current study. Fourth, a 4-point response scale was used in the current study, while a 3-point response scale was used in Kandel and Davies’ [6,13] study. Last, the current study used analytic techniques geared toward ordinal values, whereas Kandel and Davies [6,13] treated the data as continuous despite having a Likert scale with three response categories. Therefore, additional research using CFA is needed to verify whether the current findings can be replicated in other samples that vary in age, sample source (e.g., patients, community sample), and frequency of administration.

The strong associations observed between the DSS subscales are similar to those reported previously [17,51]. A frequently cited reason for the high correlations is that the etiologic origins of depression and anxiety are similar, and that anxiety and depression may co-occur [16]. However, this does not imply that these factors should be combined in measurement tools, especially since these disorders may have different determinants and/or outcomes, and they may require different treatment strategies [53]. As pointed out by Bollen and Hoyle [54], “high or even perfect correlations is not a sufficient condition to claim that a concept is unidimensional” (p. 497). In fact, a multidimensional conceptualization of the DSS may have practical utility. It could help tailor interventions when individuals have specific symptoms determined by high scores on one subscale. It could also provide key information when evaluating the effectiveness of interventions to reduce depressive, anxiety, and somatic symptoms since researchers may find change in one dimension, but not the others. This information would be masked if researchers used a combined score. Nonetheless, further work is required to increase the discriminant validity of this scale and address its other potential shortcomings, most notably that there are only two items per subscale. It is recommended that *positive affectivity* items be added to the scale (see [55] for discussion) since depression encompasses a combination of high negative affectivity and low positive affectivity, while anxiety encompasses negative aspects of affectivity only [53]. These additions may ensure that all dimensions are covered, and increase the discriminant validity of the DSS.

The factor structure of the scale was invariant across sex and time. These findings support previous research that has shown that the meaning of items measuring depression and anxiety is similar across age [28,29,56,57]. Furthermore, the findings of invariance indicate that meaningful and interpretable comparisons of mean scores can be made when using the DSS in a non-clinical sample of adolescents. However, the item uniquenesses were non-invariant across sex at Time 3, and while the item uniquenesses were invariant across time, the reliability coefficients suggest that the measurement errors associated with each item tended to be larger at the beginning of the study. This may be a result of repeated exposure to the measures which increases participants’ familiarity and reduces unrelated sources of measurement error [58]. Alternatively, the novelty of the research setting at baseline could have introduced heightened error, after which error could have decreased due to familiarity with the test protocol. Further exploration into these issues is needed. Nevertheless, it is important to note that this level of invariance is referred to as “strict invariance” and is believed to be difficult to achieve [59]. Further, it is not a requirement to test for differences in factor structure or latent means [59], and thus meaningful comparisons across sex and time can still be made when the DSS is used.

Consistent with previous research using other measures [3,4,13], individuals who scored higher on the DSS during late adolescence were more likely to report depressive symptoms in young adulthood, providing support for the predictive validity of the DSS. In contrast, the predictive validity of the DSS in regards to future anxiety disorders was relatively weak. Longitudinal studies examining the temporal relationships between depression and anxiety disorders have reported conflicting results [4,60]. One study with a community sample of youth found that depressive symptoms did not predict anxiety 3 years later [37]. In contrast, Pine et al. [6] found that a history of MDD in adolescence was significantly associated with an increase in risk for developing generalized anxiety disorder in young adulthood. As such, future research should clarify whether the onset of anxiety precedes the onset of depression, whether the onset of depression precedes the onset of anxiety, or whether there is a bi-directional association.

Limitations of this study include that only participants with complete or minimal missing data were included. While mean scores on the DSS were comparable for participants retained and not retained for the analyses, and were similar to levels reported in previous research [8], participants retained for the analyses were marginally younger than those not retained, and were more likely to be English-speaking. Though it is unlikely, it is not possible to determine if these differences biased the results. Also, although the models tested were based on theory and empirical research [19,50,51], it is possible that alternative models whereby the items load on different latent variables would adequately fit the data. Furthermore, each latent variable in the three-factor model was identified by only two indicators (i.e., items) when a minimum of three indicators per latent variable is recommended [61]. Including additional items in each factor would likely improve the reliability of the DSS and ensure that all dimensions are fully covered. Therefore, there is potential for scale improvement in future studies.

## Conclusions

The current study is the first to test alternative factor structures and the invariance of the DSS. The results enhance the utility of this brief scale by providing confirmation that it can be conceptualized as multidimensional with three related dimensions of distress (i.e., somatic, depressive, and anxiety symptoms). As such, the appropriate model to use in future studies will require careful consideration of the research question. This finding is timely in the context of the debate over the new DSM-5 which proposes the addition of a mixed depression and anxiety category. Further, both sex and longitudinal invariance of the scale were confirmed, which suggests that this scale may be used in research exploring sex differences or the development of psychological distress throughout adolescence. Accordingly, researchers seeking a short, non-burdensome measure of depressive symptoms may use the DSS in their cross-sectional and longitudinal studies to examine the links between the different dimensions of distress captured by the DSS and a range of psychological and physical health outcomes.

## Endnote

^a^Other researchers have allowed residuals for all corresponding observed variables to correlate across time points to take into account the non-independence of observations when examining longitudinal invariance. However, modification indices did not suggest that estimating these residuals in the models would improve model fit, meaning that the residuals did not correlate with each other to a statistically significant degree across time. Thus, the residuals were not allowed to correlate in the current study.

## Abbreviations

CFA: Confirmatory factor analysis; CFI: Comparative fit index; CIDI: Composite international diagnostic interview; DSM-5: Diagnostic and statistical manual of mental disorders-5; DSS: Depressive symptoms scale; GAD: Generalized anxiety disorder; ICC: Intraclass correlation coefficients; MDD: Major depression disorder; MDI: Major depression inventory; RMSEA: Root mean square error of approximation; SRMR: Standardized root mean square of the residuals.

## Competing interests

The authors declare that they have no competing interests.

## Authors’ contributions

The authors have all contributed to the submitted manuscript and support the order of authorship. Specifically, JB, CS and NL conceived the research question, JB conducted the review of the literature, analyzed data and interpreted the results, and all authors were involved in writing the paper and had final approval of the submitted version. JOL is the principal investigator of the NDIT study.

## Pre-publication history

The pre-publication history for this paper can be accessed here:

http://www.biomedcentral.com/1471-244X/14/95/prepub
